# Combination Therapy With Platelet-Rich Plasma (PRP) and Granulocyte Colony-Stimulating Factor (G-CSF) for Thin Endometrium: A Case Report

**DOI:** 10.7759/cureus.54378

**Published:** 2024-02-17

**Authors:** Sudhanshu M Dakre, Akash More, Shilpa Dutta, Shradha M Ulhe, Namrata Choudhary

**Affiliations:** 1 Clinical Embryology, Datta Meghe Institute of Higher Education and Research, Wardha, IND

**Keywords:** fet, icsi, infertility, prp, primer protocol

## Abstract

This case study highlights the challenges faced by a couple with a history of two failed in-vitro fertilization (IVF) attempts, including miscarriage and ectopic pregnancy. After treating the female partner for pelvic inflammatory disease (PID) with ceftriaxone and doxycycline, the decision was made to proceed with intra-cytoplasmic sperm injection (ICSI) and fresh embryo transfer. Despite the transfer of two good-quality (4AB and 3AA) day five embryos, the human chorionic gonadotropin (β-hCG) test yielded a negative result. Upon re-examination, a thin endometrium measuring 6.5mm was identified, prompting the implementation of the protocol for improvement of endometrial receptivity (PRIMER) protocol, which involves a combination of platelet-rich plasma (PRP) and granulocyte colony-stimulating factor (G-CSF). Following PRP administration and G-CSF injection, significant improvement was observed in the endometrial thickness. Subsequently, frozen embryo transfer (FET) was performed on day six of progesterone, resulting in a positive pregnancy outcome with a β-hCG level of 234 mIU/ml. Continuous adherence to instructions and ongoing administration of G-CSF until the 12th week of gestation remains important. This case underscores the efficacy of the PRIMER protocol in overcoming obstacles such as recurrent implantation failure (RIF) and achieving positive outcomes in assisted reproductive technology (ART).

## Introduction

Infertility is a condition in which a couple is unable to get pregnant after one year of unprotected sexual intercourse [1]. The cause of infertility is identifiable in around 85% of couples with infertility [1]. Tubal disease, ovulatory dysfunction, and male factor infertility are some of the common causes of infertility [1]. In 15% of couples, the cause of infertility remains unexplained [1]. Environmental and lifestyle modifications like smoking and obesity can affect fertility negatively [1]. Twenty-five percent of infertility cases are diagnosed with ovulatory disorders [1]. Tubal factors in females constitute a significant global public health issue [2]. Most of the cases of infertility include tubal factors that are untreated; this sexually transmitted disease causes inflammation, damage, and scarring of the reproductive tract [2].

Pelvic inflammatory disease (PID) also sometimes causes infertility; it is defined as the condition in which the upper reproductive tract in females becomes inflamed due to the infection caused by microbial pathogens [3]. In female infertility cases, around 25-35% of cases indicate issues with fallopian tubes [3]. With repeated PID episodes, the risk of tubal infertility increases; over 50% of women may experience dysfunction in their fallopian tubes after three episodes or instances [3].

Some of the common causes of PID are sexually transmitted diseases (STIs) like *Chlamydia trachomatis*, *Neisseria gonorrhoeae*, etc., which can cause tubal factors infertility (TFI) [4]. In about 30% of females, TFI is a very common cause of fertility problems [4]. STI can adversely affect pregnancy, resulting in negative outcomes [4]. The increased resistance of STI diseases like *Chlamydia trachomatis* and *Neisseria gonorrhoeae* to antibiotics is a big issue and makes these infections difficult to treat [4].

The internal lining of the uterus, called endometrium, goes through continuous variation throughout human menses. These changes, including regeneration and differentiation shedding, are orchestrated by hormones. They play a vital role in preparing the endometrium and a uterine environment, creating the ideal environment for embryo attachment (implantation) and the establishment of pregnancy [5]. Females with thin endometrial lining have a greater risk of implantation failure [6]. In patients with thin endometrial thickness or recurrent implantation failure, platelet-rich plasma (PRP) is used to improve endometrial thickness. In this type of patient, significant improvement is seen after the use of PRP [7]. Granulocyte colony-stimulating factor (G-CSF) is found to be useful in patients with thin endometrium and recurrent implantation failure. It improves endometrial thickness, and it not only increases the chances of getting pregnant but also improves the implantation rate [8]. This article is a representation of combination therapy with PRP and G-CSF for thin endometrium.

## Case presentation

Patient information

The couple, who had been experiencing primary infertility for three years, visited an assisted reproductive technology (ART) clinic situated in the rural area of the Vidarbha region. The male patient was 32 years old, while the female was 29 years old. They had undergone two failed in-vitro fertilization (IVF) cycles, with the first resulting in an ectopic pregnancy and the second ending in a miscarriage. The couple had no past surgical history, nor were they recommended to undergo any surgical procedures.

Couple medical history

The body mass index (BMI) of the male partner was 24 kg/m^2^, and that of the female was 22 kg/m^2^, demonstrating the normal BMI range for the couple. Additionally, tenderness and swelling were observed in the pelvic region of the female. The hormonal profile of the female partner is mentioned below in Table 1.

**Table 1:** Hormonal profile of the female FSH - follicle stimulating hormone, AMH - anti-Mullerian hormone, TSH - thyroid-stimulating hormone, IU/L - international unit per liter, ng/ml - nanograms per milliliter, mIU/L - milli Internation unit per liter

Hormones	Observed level	Reference levels
FSH	2.8	4.7 - 21.5 IU/L
AMH	0.6 ng/ml	1 - 4 ng/ml
TSH	0.3 mIU/L	0.4 - 2.34 mIU/L
Progesterone	8 ng/ml	10 ng/ml and more

In the HIV, hepatitis A, hepatitis B (HHH) test report, the male tested positive for gonorrhea (STI). The male partner has an asymptomatic STI. Semen analysis was performed to check the sperm count, sperm motility, and sperm morphology, all of which were observed to be within the normal range, details observed values mentioned in Table 2. A sperm chromatin dispersion test was conducted to assess sperm DNA fragmentation (SDF), and the test results were found to be normal.

**Table 2:** Details of semen analysis pH - potential of hydrogen, ml - milliliter, million/ml - millions per milliliter

Parameter	Observed limit	Reference limit
Semen volume	1.5ml	1.5 – 2.0ml
Color	Opaque white	Opaque white
Viscosity	Liquified	
Count	80mil/ml	15 million/ml or more
Progressive motility	36%	32% or more
Vitality	46%	58% or more
Normal morphology	10%	4 - 14%
pH	7.3	7.2 - 7.4
Morphological defects	85%	96% or less

**Figure 1:**
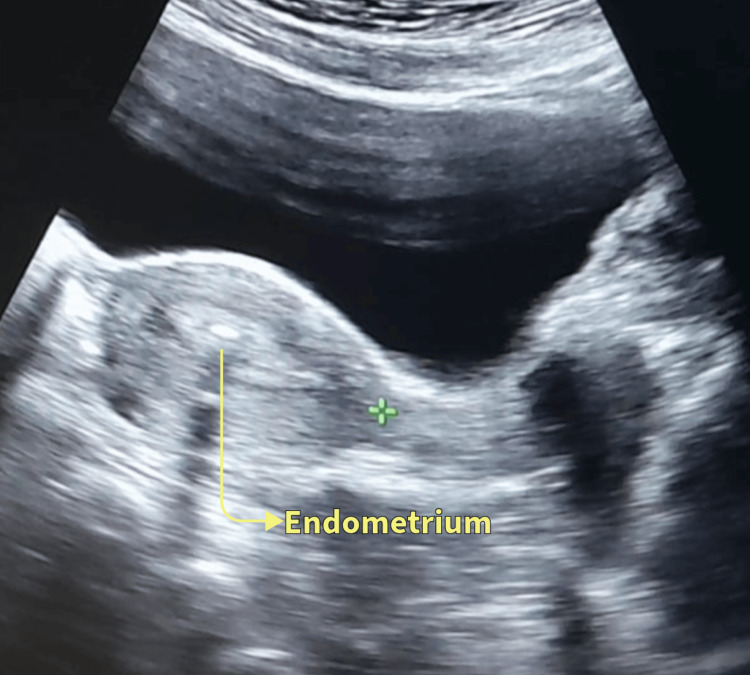
Ultrasound sonography of the female patient on day 17 of menstrual cycle

The female partners underwent specific tests to identify the cause of infertility. These tests included the follicle-stimulating hormone (FSH) test and the anti-Müllerian hormone (AMH) level test, both of which were found to be low. Endometrial thickness was checked and was observed to be below the minimum required thickness, as shown in Figure 1. Ultrasound sonography was performed to check for any structural abnormalities, and reports indicated swelling in the fallopian tube and enlarged ovaries. During the consultation, it was noted that she experienced painful intercourse, which was felt deep inside the pelvis. Since her marriage, she had experienced very painful menstruation with heavy blood flow but had not sought treatment, believing it to be normal. All these symptoms were indicative of pelvic inflammatory disease (PID). For confirmation, blood and urine tests were performed, which showed indications of gonorrhea.

Diagnosis

The couple has been facing challenges in achieving pregnancy for the first time, which indicates a case of primary infertility. Both the male and female partners were diagnosed with asymptomatic STI, and the female also had PID. Given that the female experienced painful menstruation after marriage, it can be inferred that the transmission of the STI occurred from the male to the female.

Treatment

The couple had a history of two failed IVF attempts; the first resulted in an ectopic pregnancy, and the second ended in a miscarriage. Subsequently, we planned to perform Intra-cytoplasmic sperm injection (ICSI) and fresh embryo transfer (ET). Initially, we treated the female patient for PID with oral doxycycline (100 mg) twice a day for two weeks, as doxycycline is an antibiotic used to treat STIs. Additionally, we administered a dose of intramuscular ceftriaxone (500 mg) to address STIs like gonorrhea. Following this treatment, we proceeded with ICSI as planned, scheduling ovum pickup (OPU) accordingly. The female partner underwent stimulation for OPU, and after 14 days, we performed OPU, retrieving seven good-quality metaphase II oocytes. Meanwhile, we collected a semen sample from the male partner and processed it for ICSI. The retrieved oocytes were then injected with the husband’s sperm, and the resulting embryos were cultured until day five. Prior to ET, we performed platelet-rich plasma (PRP) therapy to enhance endometrial thickness. On day five, we transferred two good-quality embryos during ET while the remaining four embryos were frozen.

Post ET, we observed that the endometrial thickness was below the required range for embryo implantation despite previous PRP treatment attempts. After consulting with the female partner, we opted to follow the PRIMER protocol and proceed with frozen embryo transfer (FET). This protocol involves the use of G-CSF and PRP to increase endometrial thickness and improve pregnancy rates in patients with recurrent implantation failure (RIF).

Accordingly, FET was scheduled. Forty-eight hours before FET, the female partner received 0.7ml of PRP through intrauterine injection, followed by subcutaneous administration of G-CSF on the same day. After 48 hours of PRP administration, the female patient underwent FET, and a significant improvement in endometrial thickness (7.5mm) was observed following the PRIMER protocol. FET was then performed, with the female partner receiving two previously frozen embryos. Following FET, the female was given rest and discharged. If pregnancy occurs, G-CSF will be continued until the 12th week of the gestational period.

Follow-up

There was no complications faced by the female throughout the procedure. The patient was advised to refrain from heavy lifting and asked to do daily core in moderation. After 14 days of FET, the female partner was called to check the human chorionic gonadotropin (β-hCG) level, and this time, it showed a positive sign for pregnancy, with a level of 234 mUI/ml. Subsequently, the female partner was monitored throughout the entire pregnancy period to ensure compliance with instructions and medications prescribed to her. Continuous administration of G-CSF was ensured until the 12th week of gestation.

## Discussion

The case study highlights the obstacles faced by a couple with a history of two failed IVF attempts, including an ectopic pregnancy and a miscarriage. To address these issues, the female patient underwent treatment for PID with doxycycline and ceftriaxone. Despite initial efforts, PRP did not yield improvement, and the fresh embryo transfer was unsuccessful. Given the negative results with PRP, we opted for the PRIMER protocol and frozen embryo transfer, combining PRP and G-CSF administration to enhance endometrial thickness. Following the PRIMER protocol, improvements in endometrial thickness were observed, demonstrating favorable outcomes. The comprehensive approach of this case, incorporating considerations for endometrial thickness, STI treatment, and the PRIMER protocol, underscores the importance of personalized strategies in overcoming challenges in ART.

In a study by Dutta et al., significant success rates with PRP were reported in younger patients [9]. Similarly, a study by Sharara et al. showed that PRP improved endometrial thickness and increased pregnancy rates in females with thin endometrium [10]. These findings informed our decision to utilize PRP in our case. Additionally, research by Dieamant et al. demonstrated the successful use of the PRIMER protocol to improve endometrial thickness [11]. Similarly, a study by Xu et al. showed significant improvements in endometrial thickness with the PRIMER protocol [12]. Consistent with these studies, our case demonstrates the successful utilization of the PRIMER protocol to enhance endometrial thickness. Hence, this study serves as a testament to the fact that the PRIMER protocol might be a valuable alternative method to treat patients with these conditions. However, since this case refers to one patient, further studies are recommended based on a larger sample population to validate the result.

## Conclusions

In conclusion, this case report underscores a couple's journey through multiple failed IVF attempts due to several problems and challenges, including miscarriage, ectopic pregnancy, and thin endometrium. Despite initial obstacles, the utilization of the PRIMER protocol, which combines PRP and G-CSF, proved promising in increasing endometrial thickness and enhancing implantation rates. This protocol was instrumental in achieving a significant improvement in endometrial thickness, enabling the successful FET procedure. This comprehensive approach highlights the importance of custom-made interventions and multidisciplinary strategies in boosting outcomes for couples facing RIF during ART. Regular monitoring and supervision remain crucial for maximizing the chances of a healthy and successful pregnancy.
